# Synergistic effects of *Ferula gummosa* and radiotherapy on induction of cytotoxicity in HeLa cell line

**Published:** 2018

**Authors:** Seyed Hamid Forouzmand, Seyed Hadi Mousavi, Vahid Vazifedan, Mahnaz Nourbakhsh, Jamshidkhan Chamani, Azar Hoseini, Azar Fanipakdel

**Affiliations:** 1 *Department of Biochemistry and Biophysics, Faculty of Sciences, Mashhad Branch, Islamic Azad University, Mashhad, Iran*; 2 *Medical Toxicology Research Center, Mashhad University of Medical Sciences, Mashhad, Iran*; 3 *Department of Pharmacology and Pharmacological Research Center of Medicinal Plants, School of Medicine, Mashhad University of Medical Sciences, Mashhad, Iran*; 4 *Cancer Research Center, Mashhad University of Medical Sciences, Mashhad, Iran*

**Keywords:** HeLa cell line, Ferula gummosa Boiss, Cytotoxicity, Apoptosis, Radiotherapy, Synergistic effects

## Abstract

**Objective:**

Cervical cancer is the second most common type of cancer among women, worldwide; and for treatment of this type of cancer radiotherapy is commonly used.* Ferula gummosa* Boiss (“Barije” in Persian, from the family Apiaceae), (F. gummosa), is an extremely precious medicinal plant which naturally grows throughout the Mediterranean and Central Asia and is a native plant in Iran. The present study examined the cytotoxic effects of *F. gummosa* in terms of induction of apoptosis and radiosensitivity in HeLa cells.

**Materials and Methods:**

In order to determine* F. gummosa *cytotoxicity in HeLa cells, the cells were incubated with different concentrations of the plant resin (0-1000 µg/ml) for 24, 48 and 72 hr. Cytotoxicity was determined by MTT assay. The role of apoptosis in *F. gummosa *cytotoxicity was investigated using flow cytometry following propidium iodide (PI) staining of DNA. For radiosensitivity assessment, *F. gummosa*-treated cells were exposed to 2 Gy γ-rays, and cytotoxicity was determined in irradiated and non-irradiated (control) groups by MTT and the synergism factor was calculated.

**Results:**

*F. gummosa* decreased cell viability in HeLa cells in a concentration- and time-dependent manner. Flow cytometry analysis indicated that apoptosis is involved in *F. gummosa*-induced cytotoxicity. Co-administration of *F. gummosa *and radiotherapy*,* showed that this plant at non-toxic low doses, could result in almost 5-fold increment in sensitization of cells towards radiation-induced toxicity.

**Conclusion:**

The concurrent use of *F. gummosa* and radiation increases radiosensitivity and cell death. Therefore, *F. gummosa *can be considered as a potential radiosensitizer agent against cervical cancer.

## Introduction

The second most common malignancy among women is cervical cancer, worldwide (Schiffman and Castle, 2005 ▶; Moody and Laimins, 2010 ▶). Clinical trials’ results have suggested chemoradiotherapy as the main treatment for women with advanced cervical cancer (Waggoner, 2003 ▶). Combination of chemotherapy and radiation can exert synergistic effects because chemotherapy may increase tumor's sensitivity towards radiation. While radiotherapy is used for local diseases, chemotherapy is used against metastatic ones (Gordon Steel and Peckham, 1979). The most commonly accepted standard therapy is the combination of surgery, ionizing radiation, and administration of multi-therapeutic agents. Anti-cancer drugs induce cell death by apoptotic or non-apoptotic mechanisms, including necrosis (Okada and Mak, 2004 ▶). The discovery and use of natural compounds as chemotherapeutic agents have attracted much attention in the treatment of cancer. In fact, a recent survey showed that the mechanism of action of most phytochemicals that act as chemopreventive drugs is mediated via change in metabolism of carcinogens, DNA repair systems suppression and reduction of cell proliferation or induction of apoptosis, leading to changes in key genomic responses. (Tayarani-Najaran et al., 2010 ▶). *Ferula gummosa* Boiss*,* a herb of the Apiaceae family, is a native plant in Iran that grows in its western and northern mountainous regions (Mortazaienezhad and Sadeghian, 2006 ▶). In traditional Iranian medicine, *F. gummosa *is used for treating different diseases. A recent chemical analysis of *Ferula* species indicated the presence of diverse phytochemicals such as sesquiterpenes, sulfur-containing compounds, coumarin derivatives and sugars among the compounds isolated. *F. gummosa *also acts as an anti-oxidant and anti-inflammatory agent (Nabavi et al., 2012 ▶; Moosavi et al., 2014 ▶). Other pharmacological effects of this plant include anticonvulsant, anti-neurological disorders, anti-diabetes, anti-rheumatic and anti-inflammatory activities. In addition, pharmacological studies have shown that *F. gummosa *resins show antibacterial, anti-catarrh, anti-microbial, antiepileptic, analgesic, digestive, carminative, aphrodisiac, laxative and expectorant properties. Moreover, the anti-hemolytic and anti-oxidant attributes of the leaf, fruit, and stem extracts of *F. gummosa *have been already reported (Nabavi et al., 2012 ▶). *F. gummosa *also exhibited a significant anti-proliferation and apoptosis-inducing effect on gastric cancer (Gharaei et al., 2011 ▶). The effect of *F. gummosa *and its radiosensitizing activity on cervical cancer have not yet been studied. The current study explores cytotoxic and apoptogenic properties of *F. gummosa*, when administered in combination with radiation, in the HeLa cell line.

## Materials and Methods

**Materials**

Propidium iodide (PI), sodium citrate, 3-(4, 5-dimethylthiazol-2-yl)-2, 5-diphenyl tetrazolium (MTT), Triton X-100, and RPMI1640 medium were obtained from Sigma Chemicals Co. (St. Louis, MO, USA). Dimethyl sulfoxide (DMSO) was purchased from Merck (Darmstadt, Germany). Fetal bovine serum (FBS) and penicillin/streptomycin were bought from Gibco (Grand Island, NY). *F. gummosa *was collected from its natural habitat in the environs of the Sabzevar, 220 km west of Mashhad, Khorasan Razavi province, Iran in June and July. The separation of the gum was performed by Mr. Amiri (a medicinal plant expert) by making three cuts in one third of the roots of each plant. After about a week, 100 to 150 g of gum was collected from each plant. A voucher specimen was submitted to the herbarium of the Faculty of Pharmacy, Mashhad University of Medical Sciences, Mashhad, Iran. 

**Cell line and cell culture**

Cervical cancer** (**HeLa**) **cells were provided by Pasteur Institute (Tehran, Iran) and kept at 37°C in a humidified atmosphere (95%) with 5% CO_2_. They were cultured in a RPMI1640 containing 10% FBS and 1% streptomycin and penicillin. The cells were grown as monolayers in a 25-cm^2^ flask.

**Cytotoxicity assessment**

*F. gummosa* cytotoxicity in Hela cells was determined using MTT assay (Mosmann, 1983; Mousavi et al., 2009). In summary, the HeLa cells were seeded at an initial density of 5000 cells/well onto flat-bottomed 96-microwell culture plates and allowed to grow for 24 hr. Next, cells were treated with different concentrations (0-1000 µg/ml) of the *F. gummosa *resin. After three different treatment time periods (24, 48 and 72 hr), the medium was removed and the HeLa cells were treated with MTT solution (5 mg/ml in PBS) for 4 hr. The resulting formazan was solubilized using DMSO (100 μl). Absorbance was read at 570 nm by an ELISA reader (Mousavi et al., 2011 ▶).

**Irradiation dose and technique**

The cultured cells were exposed to 2 Gy dose of γ radiation from a Cobalt 60 unit during exponential cell growth as monolayers in 96-microwell plates (Magne et al., 2002 ▶). During all radiation exposures, the cells were preserved in RPMI1640 medium supplemented with 10% FBS and 1% streptomycin/ penicillin. Cell viability was assessed 66 hr after radiation by MTT test (Hosseini et al., 2017 ▶).

**Radiosensitivity determination using MTT assay**

HeLa cells were cultured as described above. The cells were treated with different concentrations (0-250 µg/ml) of the *F. gummosa *resin. After 24, 48 and 72 hr of treatment, media were changed and one of the two similar plates was exposed to 2 Gy γ-rays. Then, both plates (non-irradiated and irradiated) were reincubated for 66 hr. MTT assay was carried out as previously described (Mousavi et al., 2011 ▶). Non-irradiated plates were used as resin-treated control groups. 

**Synergism factor determination**

For evaluation of radiosensitizing effects, synergism factor was calculated using the following equation: 

$\mathrm{synergism Factor}=\frac{\mathrm{Cell death induced by resin and irradiation}}{\mathrm{Cell death induced by resin}+\mathrm{Cell death induced by irradiation}}$

In cases that the resin caused cell growth instead of cell death, the cell death was considered as zero. A synergism factor>1 shows synergistic effect of the co-administration of *F. gummosa *resin and radiotherapy on HeLa cells death (Foucquier et al., 2015).

**Apoptosis assessed** **by flow cytometry**

The apoptotic cells were detected by propidium iodide (PI) staining. This was followed by flow cytometry to determine the sub-G1 peak. In brief, the HeLa cells were cultured overnight in a 24-microwell plate with an initial density of 100,000 cells/well and treated with different concentrations (30-250 µg/ml) of the *F. gummosa *resin for 48 hr. Adherent cells were then harvested and incubated at 4°C, overnight in the dark. Afterwards, the cells were washed with PBS and resuspended in 750 μl of a hypotonic buffer (50 μg/mL PI in 0.1% sodium citrate and 0.1% Triton X-100). The HeLa cells were then incubated at 37°C for 30 min before undergoing a flow cytometric analysis (Tayarani-Najarani et al., 2012 ▶). Flow cytometry was carried out by a FACScan flow cytometer (Becton Dickinson, USA). 

**Statistical analysis**

One-way analysis of variance (ANOVA) and Tukey’s *post hoc* tests were employed for several comparisons. The analysis results were expressed as mean±SEM. A p-value<0.05 was considered statistically significant. 

## Results

**Effect of synchronous radiotherapy and** ***Ferula gummosa*** **resin on Cell Proliferation**

The HeLa cells were treated with various concentrations of *Ferula gummosa *(0-250 µg/ml) for 24, 48 and 72 hr. Then, they were exposed to 2 Gy γ-rays and incubated for 66 hr. As shown in Figure 2, at low concentrations (3-30 µg/ml), *F. gummosa* did not show any significant cytotoxicity in HeLa cells*. F. gummosa* decreased the cell viability of the HeLa malignant cells in a time- and concentration-dependent manner at high doses (60-250 µg/ml). It was also found that *F. gummosa *could sensitize cells to radiation-induced toxicity at non-toxic doses (3-30 µg/ml) up to about 5 folds (Figures 2 and 3). By increasing the time of incubation from 24 hr to 48 and 72 hr, at these concentrations, the synergistic effects decreased (Figure 3). 

**Figure 1:**
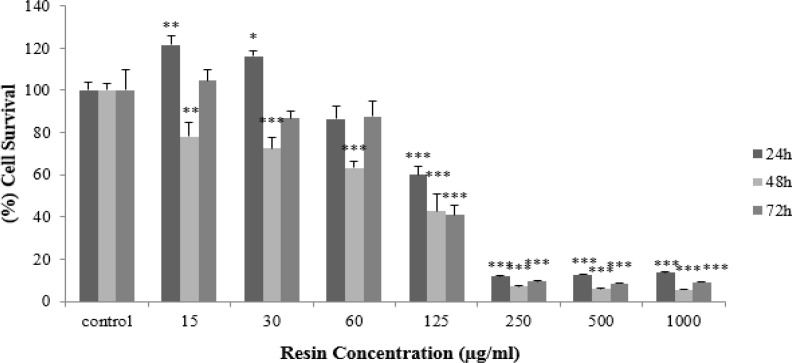
The cytotoxic effect of *Ferula gummosa* resin in HeLa cells. The cells were treated with 0-1000 µg/ml concentrations of *F. gummosa* for 24, 48 and 72 hr. Cell survival was quantitated by MTT assay. The data are expressed as mean±SEM (n=3). *p<0.05, **p<0.01, and ***p<0.001 show significant differences as compared to the control group.

**Figure 2:**
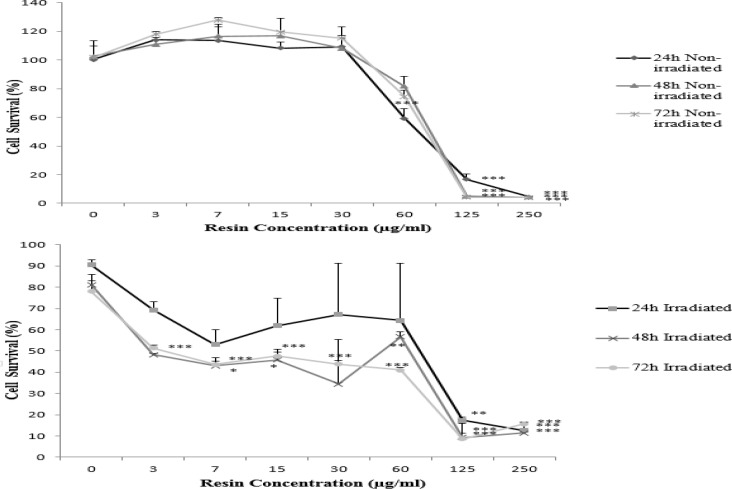
Comparison of the cytotoxic effect of different concentrations of *Ferula gummosa *and its radiosentisizing effects on HeLa cells. Two groups of HeLa cells were treated with various concentrations of *F. gummosa *for 24 hr (A), 48 hr (B), and 72 hr (C) and one of two groups was exposed to 2 Gy γ-rays. Both groups (non-irradiated and irradiated) were incubated for more 66 hr. Viability was quantitated by the MTT assay. The data are expressed as mean±SEM (n=3). The asterisks indicate statistical differences when compared to the control of each group (Resin concentration=0 μg/ml) represented in the Figure as *p<0.05, **p<0.01, and ***p<0.001.

**Figure 3:**
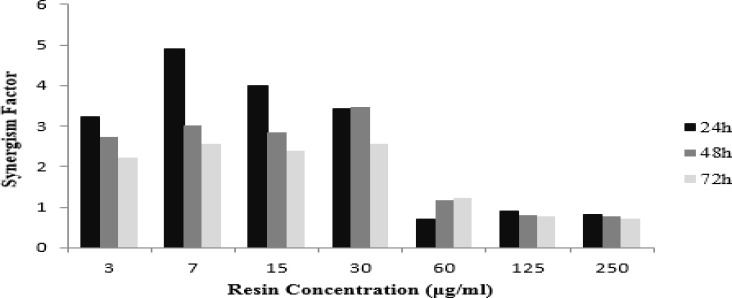
Synergistic effects of co-administration of different concentrations of *Ferula gummosa *resin and radiotherapy in HeLa cells. Cells were treated with various concentrations of *F. gummosa *Boiss for 24, 48 and 72 hr. One group received 2 Gray of γ-rays and the other one was used as control group. MTT assay was carried out after 66 hr. Synergism factor was defined as relative combination therapy cell death to sum of cell death caused by individual resin and irradiation at each time point and concentration. A synergism factor>1 shows synergistic effects of combination therapies.

**Apoptosis induction by** ***Ferula gummosa*** **resin in HeLa cells**

**Figure 4:**
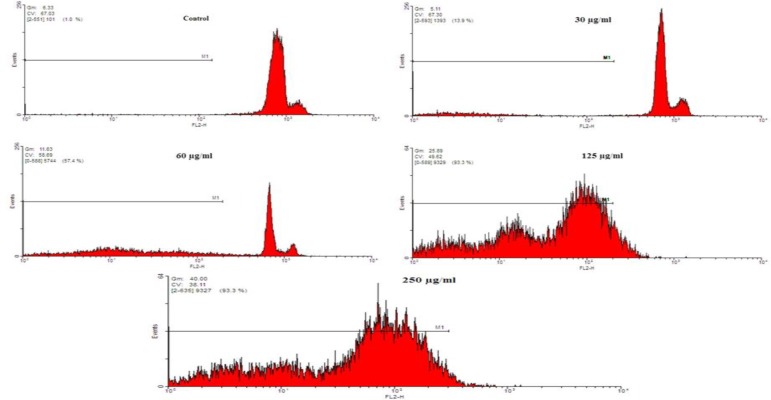
The role of apoptosis in *Ferula gummosa-*induced toxicity in HeLa cells. HeLa Cells were treated with 30-250 µg/ml of *F. gummosa *for 48 hr. Apoptosis was assessed by flow cytometry following propidium iodide staining. A sub-G1 peak, as an indicator of apoptotic cells rate, was induced in the *F.* gummosa-treated cells but not in the control cells.

**Figure 5:**
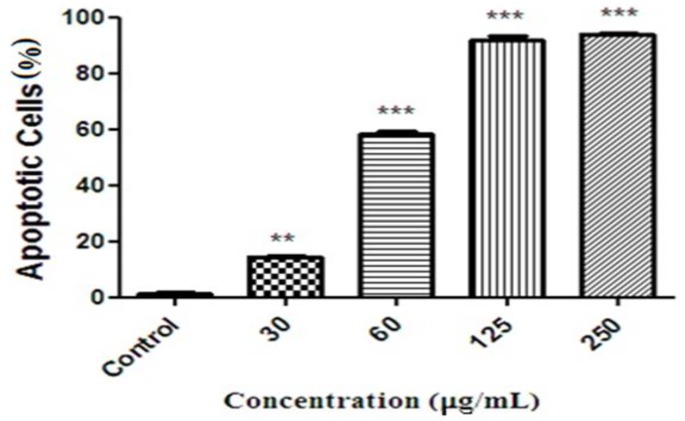
The percentages of apoptotic cells in the HeLa cells treated with different concentrations of* Ferula gummosa*. HeLa Cells were treated with 30-250 µg/ml of *F. gummosa *for 48 hr. The percentages of apoptotic cells were determined by flow cytometry histograms. Results are demonstrated as mean±SEM of three independent experiments. Statistical differences are shown as **p<0.01, and ***p<0.001, as compared to control.

After treatment of the HeLa cells with* F. gummosa *resin (30-250 µg/ml), apoptosis induction was investigated by PI staining of DNA followed by flow cytometry evaluation. In the flow cytometry histogram of the treated cells (Figure 4), a sub-G1 peak was observed when compared with the control. The flow cytometry histogram confirmed the induction of apoptosis in the induced toxicity. Results displayed that *F. *gummosa induced apoptosis in a concentration-dependent manner. The percentages of apoptotic cells in the HeLa cells are shown in Figure 5.

## Discussion

Plants extracts as natural products have been found effective for treatment of several disorders (Ouyang et al., 2014 ▶). The genus *Ferula* belongs to the Apiaceae family. This genus contains a lot of biologically active components that sound to be suitable if used as adjuvants in disease treatments (Iranshahi et al., 2018 ▶). Cytotoxic effects are one of the main biological activities of the genus *Ferula*. *F. gummosa* and *F. assa-foetida*, the two popular species of *Ferula* in Iran. 

 In 2016, Gharaei *et al* showed that *F. gummosa* Boiss. extracts suppressed gastric cancer cells’ proliferation through apoptosis induction (Gharaei et al., 2013 ▶). Gudarzi et al. also showed that ethanolic extract of *F. gummosa* seeds induce apoptosis in BHY (human oral squamous cell carcinoma) cell line (Gudarzi et al., 2015 ▶). Additionally, cytotoxic effects of hydroalcoholic extract of *F. gummosa* root have been shown in GP -293 cell line and primary cultured human stromal -vascular cells (Ghorbani et al., 2016 ▶). These studies suggested *F. gummosa* extracts as a chemotherapeutic agent, but there was no information available on possible radiosensitizing effects of *F. gummosa*.

The concurrent use of chemotherapy and radiotherapy in the cervical carcinoma cell line, has been studied in several works. Yang *et al*. analyzed the effects of CXCL10, in combination with radiation, in the cervical cancer cell line. They demonstrated that the combination of CXCL10 and radiotherapy effectively inhibited the growth of the cervical carcinoma cell line (Yang et al., 2012). Luo *et al*. examined the effect of a combination of a drug (an artemisinin derivative) and radiation in cervical cancer cells. They found that the combination treatment increased apoptosis in HeLa cells (Luo et al., 2014 ▶). In a study on 19 human cervical cancer cell lines, Britten *et al*. discovered that when radiotherapy is co-administered with cisplatin, the death rate of these tumor cells increases (Britten *et al.,* 1996). Rose et al. demonstrated that concurrent treatment using radiotherapy and chemotherapy (with cisplatin) improves the rates of survival and progression-free survival among women with locally advanced cervical cancer (Rose et al., 1999 ▶). Since no information was available on the effects of *F. gummosa* resin combined with radiotherapy, the present study determined the effects of *F. gummosa*, combined with radiation, against the HeLa cell line. 

In this study, first, we evaluated the effects of *F. gummosa* Boiss. on HeLa cells to gain more insight into the effects of this plant on the induction of cell death and inhibition of cell proliferation. Cell viability was quantified by the MTT assay and the results revealed that *F. gummosa* exerts cytotoxicity activity in this cell line. *F. gummosa* decreased cell viability in HeLa cells in a time- and concentration-dependent manner. Furthermore, *F. gummosa-*induced apoptosis was involved in the induction of cell death. For many years, it has been known that many therapeutic factors kill cancer cells by inducing apoptosis (Debatin et al., 2002 ▶; Hersey and Zhang, 2001 ▶, Mousavi et al., 2008 ▶). Compared to the control, *F. gummosa* induced a sub-G1 peak in the flow cytometry histogram of treated cells, suggesting that apoptotic cell death is involved in its toxicity*.*

 At the next step, the effects of a combination of *F. gummosa* and radiation against the HeLa cells, were determined. The results confirmed that concomitant use of them increases radiation sensitivity and radio response of the HeLa cell line, leading to increased cell death. We observed synergistic effects at non-toxic low doses of *F. gummosa *when co-administered with radiotherapy that suggest it a suitable radiosensitizer*.* Dayal et al. suggested that radiation‑induced reactive oxygen species (ROS) production play an important role in the tumor cell killing by triggering apoptosis. Furthermore, they pointed that concurrent use of plant‑derived antioxidants and radiation lead to ROS over-production in tumor cells thus enhances radiosensitivity (Dayal et al., 2014 ▶). As *F. gummosa is* a rich source of natural antioxidants (Nabavi et al., 2012 ▶), we think that this mechanism may play a key role in the radiosensitizing activity of *F. gummosa* observed in this experiment. 

This investigation is the first to present the synergistic effects of *F. gummosa* and radiotherapy on Hela cells. This preliminary study might be beneficial for developing a new approach to be used in clinical settings.

*F. gummosa* sensitizes cells towards radiation-induced toxicity, in which apoptosis plays a significant role. It can also be considered as a promising chemotherapeutic drug for cervical cancer treatment.
